# A global association between Covid-19 cases and airborne particulate matter at regional level

**DOI:** 10.1038/s41598-021-85751-z

**Published:** 2021-03-18

**Authors:** Angelo Solimini, F. Filipponi, D. Alunni Fegatelli, B. Caputo, C. M. De Marco, A. Spagnoli, A. R. Vestri

**Affiliations:** 1 Department of Public Health and Infectious Diseases, Sapienza University of Rome, Piazzale Aldo Moro 5, 00185 Rome, Italy; 2 Institute for Environmental Protection and Research (ISPRA), Via Vitaliano Brancati 48, 00144 Rome, Italy

**Keywords:** Viral infection, Risk factors

## Abstract

Evidences of an association between air pollution and Covid-19 infections are mixed and inconclusive. We conducted an ecological analysis at regional scale of long-term exposure to air-borne particle matter and spread of Covid-19 cases during the first wave of epidemics. Global air pollution and climate data were calculated from satellite earth observation data assimilated into numerical models at 10 km resolution. Main outcome was defined as the cumulative number of cases of Covid-19 in the 14 days following the date when > 10 cumulative cases were reported. Negative binomial mixed effect models were applied to estimate the associations between the outcome and long-term exposure to air pollution at the regional level (PM_10_, PM_2.5_), after adjusting for relevant regional and country level covariates and spatial correlation. In total we collected 237,749 Covid-19 cases from 730 regions, 63 countries and 5 continents at May 30, 2020. A 10 μg/m^3^ increase of pollution level was associated with 8.1% (95% CI 5.4%, 10.5%) and 11.5% (95% CI 7.8%, 14.9%) increases in the number of cases in a 14 days window, for PM_2.5_ and PM_10_ respectively. We found an association between Covid-19 cases and air pollution suggestive of a possible causal link among particulate matter levels and incidence of COVID-19.

## Introduction

It is well known that exposure outdoor air pollution—and in particular to particulate matter PM (PM_10_, PM_2.5_), to nitrogen oxides (NO and NO_2_), as well as to ozone (O_3_)—can have a variety of adverse health effects that include, but are not limited to, cardiovascular diseases^1,2^, asthma^3,4^, chronic obstructive pulmonary disease (COPD)^5,6^, liver diseases^7^, diabetes^8^ and cancer^9^. Recently, particular attention was devoted to potential effects of fine particulate matter (PM) on the initial spread of the epidemic and the prognosis of the disease in Covid-19 patients. The underlying hypothesis is that higher concentration of PM makes (a) easier to the virus entering in the respiratory system, and its long-term exposure makes (b) the organism more susceptible to the infection and its complications, once infected.

In exposed individuals, airborne particles may directly increase the vulnerability to Covid-19 by serving as carrier of viral RNA^10^ and indirectly by increasing the effects of the virus on the lungs^11^. Air pollutants might therefore be a sort of “co-factors” of indirect systemic effects causing pro-inflammation and oxidation mechanisms in the lungs (and extrapulmonary organs) and alteration of the immune system. The possible interaction between pollution levels and Covid-19 is also suggested by the fact that exposure to air pollution increases the risk of influenza like illness, respiratory diseases and acute lower respiratory tract infections in vulnerable individuals^12,13^*.* Therefore, it may be possible that the higher and prolonged over time is the exposure to PM (as for the elderly), the higher is the probability that the respiratory system is vulnerable to more serious consequences of the infection.

To date most analysis published so far examined the number of Covid-19 cases or deaths and long term exposure of air pollution within the same country^11,14–16^. Those studies were therefore limited by the air pollution range exhibited by the administrative units within a given country restricting the external validity of the results. Additionally, most early analyses were not adjusted by non-pharmaceutical interventions, that varied widely between countries in both timing and severity or were not comparable in terms of other covariates used to adjust the statistical models. Taken together, available evidences from published studies are mixed and critically different in terms of statistical models, covariate adjustment, outcome and exposure definitions and geographical units.

We conducted a worldwide ecological analysis to evaluate the hypothetical association between reported Covid-19 cases in early phase of epidemics and long-term exposure to particulate matter air pollution at the subnational (i.e. regional) level. Within each region, pollution levels were computed in the most populated area at the unprecedented 10 km resolution and several relevant covariates were included at regional and country levels.

## Methods

This analysis is based on a large global dataset built by collecting information from various freely available sources up to May 30, 2020. The geographic dataset for region-equivalent areas at full global extent was populated with demographic, climatic, air quality data and Covid-19 time series of cases. Additionally, we added several country level covariates including cumulative number of tests, Government Response Stringency Index^17^, prevalence of diabetes and gross domestic product as detailed below.

### Administrative units and spatial variables

Region-equivalent administrative units were collected from GADM version 3.6 (https://gadm.org), mapping spatial polygons of administrative areas for all countries of the World, at all levels of available sub-division. NUTS spatial polygons at level 2, corresponding to the region spatial polygons of the European countries, were downloaded from Eurostat (https://ec.europa.eu/eurostat/web/nuts/nuts-maps). Each administrative unit was uniquely identified by a code and for each polygon the latitude and longitude coordinates of the polygon centroid were calculated. In order to take into account the spatial autocorrelation within models, avoiding the use of latitude information that is likely correlated to climate covariates (i.e. temperature), distance (d) and bearing (b) from the origin (Longitude = 0°, Latitude = 0°) have been computed from each region centroid coordinates as follows:

$$d = \sqrt {Longitude^{2} + Latitude^{2} }$$

$$b = \frac{180}{{\pi *atan2\left( {Longitude,Latitude} \right)}}$$

where atan2 is a four-quadrant inverse tangent requiring two input values (Longitude and Latitude).

### Demographic variables

Each administrative unit was linked to information about population counts, population density, sex and age structure. Population counts and densities were derived from the Gridded Population of the World (version 4.11, distributed by Socioeconomic Data and Applications Center), which provides spatially disaggregated population datasets^18^ for year 2020. Population counts are defined at a horizontal grid spacing of 30 arc seconds (approximately 1 km × 1 km at the equator) and were used to compute spatial statistics for each administrative unit. Population sex and age structure were calculated from the basic demographic dataset, which is available for year 2010. Finally, a 10 × 10 km spatial kernel was used to identify geographic coordinates of the location with the higher population size within each administrative unit.

### Climate data

Climate datasets have been collected from the Copernicus Climate Change Service (C3S) (https://climate.copernicus.eu). We downloaded “2-m temperature” and “2-m dewpoint temperature” variables from ERA5 single levels reanalysis dataset^19^ for the period November 2019–May 2020. ERA-5 reanalysis climate data are provided at hourly time step, on a spatial grid with 0.25° horizontal resolution (approximately 30 km × 30 km at the equator), covering the entire Earth. For each grid cell we calculated daily mean, maximum, minimum for the variables 2-m air temperature, relative humidity and absolute humidity. The 2-m temperature is the temperature at the height of 2 m above Earth’s surface. Relative humidity is the percentage of the maximum amount of water vapor that the atmosphere can hold at a given temperature (saturation). Relative humidity has been calculated from 2-m temperature and 2-m dewpoint temperature, according to the formula in^20^. We extracted daily statistics on temperature and relative humidity from the cell spatially closest to the geographic coordinates of the location with higher population size within each administrative unit.

### Air quality

Air quality datasets were collected from the Copernicus Atmosphere Monitoring Service (CAMS) (https://ads.atmosphere.copernicus.eu). The ECMWF Atmospheric Composition Reanalysis 4 (EAC4) global reanalysis product^21^ are provided at three hourly time step, on a spatial grid with 0.75° horizontal resolution (approximately 90 km × 90 km at the equator), covering the entire Earth. Selected variables are the concentration (μg/m^3^) of PM_2.5_ and PM_10_ at the Earth surface. For each grid cell we calculated daily mean, maximum, minimum and percentiles statistics for the period 2014–2018 and daily statistics of PM_2.5_ and PM_10_ were extracted from the cell spatially closest to the geographic coordinates of the location with higher population size within each administrative unit. CAMS ensemble estimates may have a bias (quantified as temporal root mean square error) around 3.0 μg/m^3^ for PM_2.5_ and around 4.3 μg/m^3^ for PM_10_.

### Covid-19 cases

The Covid-19 dataset was built by integrating several repositories on Github, that collected regional daily cases by country up to May 30, 2020. Most of the sources were official reporting bodies, such as the EC- Joint research Centre for European countries (https://github.com/ec-jrc/COVID-19). The sources of data for other world regions are listed in supplementary Table S1 and at this link (https://tinyurl.com/yxmh6blb). The sum of reported cases by country was almost identical to WHO official country level data (see supplementary figure S1). Daily cases at regional level were then linked to GADM administrative areas codes (https://gadm.org/) and NUTS_2 administrative areas codes (for European countries, https://ec.europa.eu/eurostat/web/regions-and-cities/overview).

### Covariates at country level

Information on the Covid-19 cumulative tests performed at the date of outcome were extracted from our world data (https://ourworldindata.org/) or from official ministry websites. For Andorra, Bosnia-Herzegovina, Belarus, China, Algeria, Makedonia and Montenegro, we conservatively imputed the total number of tests to be equal to the country level cumulative number of cases. As indicator of country response to the Covid-19 crisis we used the Government Response Stringency Index^17^ that is a composite measure based on nine response indicators including school and workplace closures and travel bans, rescaled to a value from 0 to 100 (100 = stricter response). We extracted country level pro-capita gross domestic product (in current US$), that is a proxy of health-related infrastructures and more (life expectancy, infant mortality, etc.), and prevalence of diabetes from world bank database (https://data.worldbank.org/indicator).

### Outcome definition and statistical analysis

From each Covid-19 case time series, we derived an indicator of initial outbreak spread in each administrative unit that could be used as outcome in the statistical analysis. The main outcome was defined as the cumulative number of cases in the 14 days following the date when > 10 cumulative cases were reported. We also calculated a more conservative outcome that were defined as the cumulative number of cases in the 14 days following the date when > 50 cumulative cases were reported.

We used negative binomial mixed models to estimate the association between the outcome and long-term exposure to air pollution. The primary analysis was performed considering the main outcome defined above as the dependent variable and PM_10_ or PM_2.5_ as fixed effects. For each region, summary statistics of daily temperature and RH were computed as mean over the 30 days before each outcome date while chronic exposure to PM_2.5_ and PM_10_ as averages of mean daily levels over 2015–2018.

The analysis was adjusted by several covariates at administrative unit level (total population, proportion of total population aged 65 or up, sex, temperature, relative humidity) and at country level (diabetes prevalence, gross domestic product per individual, cumulative number of tests at outcome date, Government Response Stringency Index at outcome date). This final set of covariates was selected after the usual preliminary inspection of correlation structure and fit of each variable in turn in the model. A random intercept by country was included to account for potential correlation in regions within the same state, due to similar sociocultural, behavioral, and healthcare system features and similar Covid-19 response and testing policies. To control for the spatial structure of data we included in the model natural cubic splines of distance and bearing as computed above (see “Administrative units and spatial variables”). The analysis was repeated also adjusting for country as fixed effect, to capture eventual remaining differences in the number of cases between countries not explained by fitted exposure and covariates.

Sensitivity analyses were applied to assess the robustness of results to confounders by fitting the full models without a single covariate in turn and comparing model goodness of fit by the Akaike information criterion (AIC). All of the statistical analyses were performed using R version 4.0.2.

## Results

**Figure 1:**
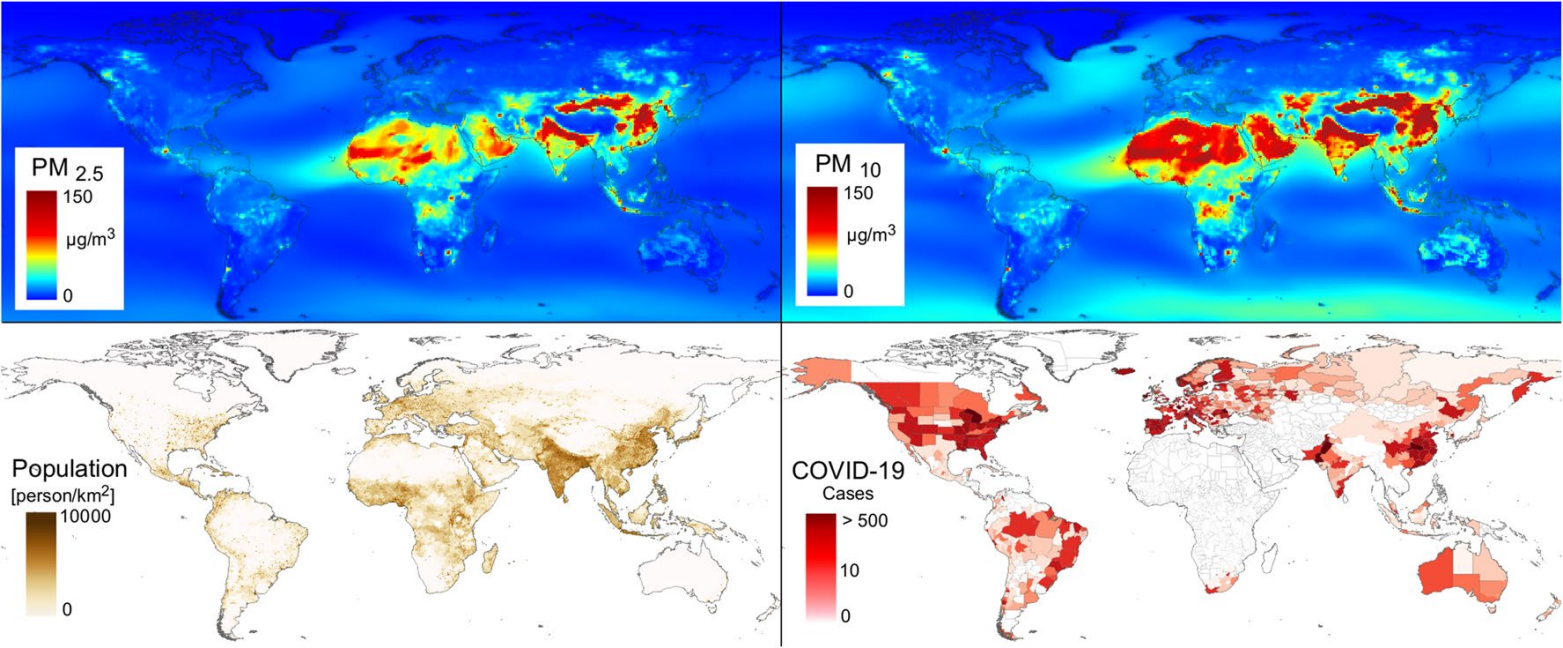
Airborne fine particulate matter average levels (μg/m^3^) population densities and Covid-19 daily cases in the 14 days after outcome date (date when cumulative number of cases = 10; up to May 30, 2020). PM levels refer to mean daily concentrations over 2015–2018.

In total, we collected 237,749 Covid-19 cases from 730 regions from 63 countries and 5 continents at May 30, 2020 (Fig. 1, Table 1). The date of the main outcome (cumulative number of cases in the 14 days following the date when > 10 cumulative cases were reported) varied between February 10 (China) and May 25 (Bolivia) and the number of daily cases in the 14 days window varied between 0.78 and 2116 (Fig. 1). Considering all regions, in the 30 days before the outcome date the average temperature ranged between min = 3.6 °C and max = 30.7 °C (median = 13.3 °C), relative humidity between 51.3 and 84% (median = 70.2%). Average airborne fine particulate matter (PM_2.5_) ranged between 3.3 and 263.5 μg/m^3^ (median = 13.1 μg/m^3^), while PM_10_ ranged between 3.5 and 372.0 μg/m^3^ (median = 33.3 μg/m^3^). At time of outcome most of the countries had government containment measures (ie. Government Response Stringency Index) in place (mean SI = 76.7) and reported a cumulative total of 242,910,857 Covid-19 tests.

**Table 1:** Environmental, socio-demographic and Covid-19 median and interquartile range.

Variable	Median (IQR)
Covid-19 daily cases in the 14 days after outcome date	9.07 (4.43; 23.71)
Outcome date (cumulative number of cases = 10)	03-04-2020 (26-03-2020; 15-04-2020)
Total population in the most populated area of the region	1,648,510 (935,028; 3,826,890)
Proportion of population 65 +	0.11 (0.06; 0.14)
Proportion of male population	0.49 (0.48; 0.50)
Diabetes prevalence (%)	6.6 (6.1; 9.3)
Cumulative number of tests	64,387 (34,938; 208,887)
Stringency index	76.85 (72.69; 85.19)
Per capita annual GDP ($)	12,238 (8,717; 40,493)
PM_10_ (μg/m^3^)	33.29 (14.18; 25.77)
PM_2.5_ (μg/m^3^)	13.07 (9.2; 18.28)
Temperature (°C)	13.31 (9.93; 22.22)
Relative humidity (%)	70.23 (62.19; 77.67)

Cumulative number of Covid-19 tests, Government Response Stringency Index at outcome date; temperature and relative humidity in the 30 days before the outcome date. PM levels refer to mean daily levels recorded over 2015–2018.

Regional mean number of Covid-19 cases were positively associated with air pollution levels (Table 2) in fully adjusted models. A 10 μg/m^3^ increase of PM_10_ was associated with 8.1% (95% CI 5.4%, 10.5%) increase in the number of and 10 μg/m^3^ increase of PM_2.5_ was associated with a 11.5% (95% CI 7.8%, 14.9%) increase in the number of Covid-19 cases in the 14 days following the date when > 10 cumulative cases were reported (i.e. Outcome_10, in Table 2). Estimates of this model were robust to different definition of the outcome or when we entered the country as fixed effect in the model (Table 2). In sensitivity analysis, pollutant estimates remained similar when we removed a single covariate in turn (Table 3).

**Table 2:** Association between long term exposure to particulate matter and Covid-19 cases at regional level estimated with negative binomial regression models with country entered as random (mixed effect model) or fixed (fixed effect model).

Model	Pollutant	Outcome_10	AIC	Outcome_50	AIC
Mixed effect	PM_10_	1.008(1.005, 1.011)	7949.7	1.007(1.005, 1.009)	7470.2
	PM_2.5_	1.012(1.008, 1.015)	7949.2	1.010(1.007, 1.013)	7469.2
Fixed effect	PM_10_	1.008(1.005, 1.011)	7909.8	1.007(1.005, 1.010)	7412.8
	PM_2.5_	1.012(1.007, 1.016)	7909	1.010(1.007, 1.014)	7411.7

Models adjusted for total population, proportion of population aged 65 or up, sex, temperature, relative humidity, diabetes prevalence, gross domestic product per individual, cumulative number of tests at outcome date, Government Response Stringency Index at outcome date and spatial components entered as cubic splines. Outcomes are the cumulative number of cases in the 14 days following the day when the 10th (Outcome_10) or 50th (Outcome_50) case were reported in each region. Reported values are the exponentiated betas and 95% confidence interval.

**Table 3:** Sensitivity analysis: coefficients (exponentiated betas, 95% confidence interval) of associations between main outcome (Covid-19 cumulative number of cases in the 14 days following the date when > 10 cumulative cases were reported) and air pollutants in models adjusted by all but one variable in turn.

Removed variable from the full model	PM_10_	AIC	PM_2.5_	AIC
Total population	1.011 (1.009, 1.014)	7964.7	1.016 (1.013, 1.020)	7968.4
Per capita annual GDP ($)	1.007 (1.005, 1.010)	8070.6	1.010 (1.007, 1.014)	8070.2
Stringency index	1.008 (1.005, 1.011)	7976.8	1.012 (1.008, 1.015)	7976.3
Cumulative number of tests	1.008 (1.005, 1.010)	9179	1.011 (1.007, 1.014)	9178.4
Proportion of population 65 +	1.008 (1.006, 1.011)	7954.2	1.012 (1.008, 1.015)	7953.7
Sex	1.008 (1.005, 1.010)	7963.4	1.011 (1.007, 1.014)	7962.9
Diabetes prevalence	1.010 (1.008, 1.013)	8034.9	1.012 (1.008, 1.015)	7949.5
Temp and rh	1.008 (1.005, 1.011)	7951.8	1.012 (1.008, 1.015)	7951.3
Spatial components	1.008 (1.005, 1.010)	7961.7	1.011 (1.007, 1.015)	7961
None	1.008 (1.005, 1.011)	7949.7	1.012 (1.008, 1.015)	7949.2

Temp and rh: temperature and relative humidity in the 30 days before the outcome date; spatial components: cubic splines of distance and bearing. Reported values are the exponentiated betas and 95% confidence interval.

## Discussion

We compiled a global database of Covid-19 cases referred to the first wave of the pandemics up to May 30, 2020 and studied the association with climate and pollution statistics referred to the most populated areas within each region, regional demography and other covariates at country levels for 730 regions belonging to 63 countries and 5 continents. We found an increase between 8.1 and 11.5% in the number of Covid-19 cases for a 10 μg/m^3^ increase of PM_10_ and PM_2.5_, respectively, after controlling for important spatial area level covariates. Significantly positive associations of air pollutants with Covid-19 confirmed cases or deaths were already reported for China^22^, Italy^23^, Canada^24^, USA^25^, Japan^26^, Netherlands^27^, UK^14^ and regions across Italy, France, Germany, and Spain^15^. However, to our knowledge this is the first paper examining the association between Covid-19 spread and pollution worldwide, controlling for a wide range of regional and country potential confounding effects. The compelling evidence of a statistically significant positive relationship between air pollution and Covid-19 cases was robust to changes of predictors included in the model. Still, not including in the statistical model total population residing in the area increased the PM_10_ effect to 11% and PM_2.5_ to 16%, pointing to a not surprising covariation of pollution levels and size of resident population. Therefore, most populated and polluted (i.e. urban) areas worldwide are possibly places where most sustained virus transmission takes place and where quickly increasing hospitalizations are expected.

The association between air pollution and Covid-19 spread was reported in analyses conducted in several countries. Studies published so far were manly cross-sectional by design (e.g. conducted at aggregate level) and varied in terms of geographical unit examined (from regions to provinces to states), time span of exposure assessment (short term or long term), metric of air pollution and in the number of covariates used in adjusting the statistical models.

A cross-sectional analysis was conducted in 120 cities (4 municipalities and 116 prefecture-level cities over a period between final Jan-2020 to final Feb-2020) of China^22^. The authors found an increase of 2.24% (95% CI 1.02–3.46) and 1.76% (95% CI 0.89–2.63) in the daily counts of Covid-19 confirmed cases for an increase of 10-μg/m^3^ (at cumulative lag of 0 -14 days) in PM_2.5_ and PM_10_, respectively^22^. Similar results were found at the Province level in Italy by several authors^23,28,29^. In contrast, in 28 geographical areas of Japan^26^, the epidemic growth (as cumulative count of confirmed cases over a period March—6 April 2020) was not found statistically associated with PM_2.5_ (and other pollutants), after adjusting for demographic variables.

Long term exposure effect of particulate matter was also studied in several countries. A study in Canada^24^ examined 111 health regions up to May 13, 2020 using long term exposure to air pollution. Data were analyzed using multivariate regression in which the incidence of Covid-19 cases was modelled as function of long-term exposure of PM_2.5_, and other fixed effects such as provinces and other ecological/social parameters (such as temperature, demographic and health characteristics), added as controlling factors. The incidence rate was not statistically significant in the main analysis (incidence rate = 1.07, 95% CI 0.97, 1.18 per a unit increase of pollutant). However, a positive and statistically significant association was found when restricting the analysis to the most Covid-19 affected provinces (Quebec and Ontario). Similar results were found when regressing air pollution of 55 Italian cities (measured with days exceeding the limits set for PM10 over 2018 year) and number of Covid-19 cases (up to 7th April 2020)^30^. Moreover, a national-wide cross-sectional study was conducted in USA^25^, using more than 3000 county up to 22 April 2020, fitting a negative binomial regression model. They found an 8% increase in the Covid-19 mortality rate (95% CI 2%, 15%) per unit increase of PM_2.5_. Remarkably, the model was adjusted for wide range of potential confounders (i.e. socioeconomic, demographic, weather, behavioral, epidemic stage, and healthcare-related) and was robust to sensitivity analysis. A similar study was conducted in 355 relatively small Dutch municipalities^27^ using as outcomes the count of infected, hospitalized and death cases of Covid-19 (data up to 5th June 2020) and as long term exposure the annual concentrations averaged over the period 2015–2019 of PM_2.5_. Using a negative binomial regression model, adjusting for different confounding effects (demography, social and physical proximity, employment, education, spatial and health variables), the study revealed statistically significant positive relationship between air pollution and Covid-19 cases, finding an increase of 9.4 more Covid-19 cases, 3.0 more hospital admissions, and 2.3 more deaths per unit increase of PM_2.5_.

An important strength of our study is that it integrates Covid-19 global epidemic data with an extensive set of spatially explicit covariates related to socio-demographic, climatic, air quality and environmental variables at fine spatial scale. The geographic database populated with up-to-date datasets at full global extent, also including products distributed by the services developed under the European Copernicus program, allowed to conduct a statistical analysis at an unprecedent spatial detail, revealing the implications of diverse factors in the epidemic spread. It should be noticed that although minor biases in the air pollution estimates using the CAMS ensemble median datasets might be present, spatial associations with response variables should be unbiased. The estimates of the pollution effects obtained from mixed models remained the same in fixed effect models, pointing to the fact that most of the variation in the response variable is probably due to differences between countries^31^. Our findings have also implication for preparedness to future waves of Covid-19 or other respiratory viruses. It appears urgent to understand the role played by multiple environmental and socio-economic variables that could confound or possibly modify the association between air pollution and epidemic spread. The design of collaborative observational studies using detailed individual-level data on Covid19 cases coupled with environmental exposures should therefore be prioritized. However, in the view of the precautionary principle, the partial or incomplete evidence already provided by observational studies published so far should be considered by policy makers, without waiting for further confirmation.

This study has also several limitations. We inferred an ecological association and we could not make any inference about causal relationships. Although our findings are correlative, several plausible mechanisms through which the long term exposure to air pollution might affect Covid-19 spread and mortality were reported^16^. Air pollution is associated with increase inflammatory cascade in exposed subjects and consequent overstimulation of the immune system. Chronic inflammation might therefore increase the susceptibility of exposed population to Covid-19, increasing the risk of Covid-19 related hospitalization. Since the link between air pollutants and other respiratory viruses was already reported in the literature^13^, it is plausible that areas were more population is exposed to the effect of air pollutants are also the areas were faster spread of Covid-19 cases are reported. Second, we attributed exposure levels on a geographical basis at the lower geographical resolution scale that was possible, given the global nature of our effort. This may lead to exposure misclassification as we are assuming that air pollution levels in a specific area are representative of the real long-term exposure of each individual living in that area. Third, bias in reported number of cases, especially in regions where the health system was unprecedently under pressure, might also affect this analysis. However, the results were substantially the same when we used as outcome the number of cases in the two weeks following the day when 50 cases (rather than 10) were recorded that implies also a time window shift and a different attribution of exposure level. Fourth, the exclusion of potentially important covariates that were not available at regional level (e.g. number of tests) might also affect our analysis. For some important covariate like cumulative tests performed or population diabetes prevalence we could use the country level (i.e. aggregated) value in the statistical models. It should also be remarked that in our statistical models all variables had little influence on the pollution term, with the exception of population density. Also including country as fixed and not random effect, and therefore capturing all residual variance associated with between country differences, caused a small decrease of the pollution term. Still, the association between Covid-19 cases and PM levels remained positive and above the null.

## Conclusion

This study investigated the effects on Covid-19 infections of PM_2.5_ and PM_10_ after adjusting for temperature, relative humidity and other socio-demographic and economic covariates. We found an association between Covid-19 cases and pollution level suggestive of the existence of a possible causal link among those two factors. This finding has obvious implications for policymakers, clinicians and public health authorities. The burden of Covid-19 cases due to air pollution represent an additional cost in terms of population health that might be avoidable by reducing pollution levels.

## Supplementary Information

Supplementary Information

## Data Availability

All data used in this manuscript is publicly available. An automated linux bash script for downloading data and build our dataset will be available at the gitHub repository https://github.com/randomxsk8/covid19_regional.
