# Supplementary material for: A global association between Covid-19 cases and airborne particulate matter at regional level
Source: Sci Rep. 2021 Mar 18;11:6256. doi: 10.1038/s41598-021-85751-z (PMC7973572; doi:10.1038/s41598-021-85751-z)
Supplement: Supplementary file 1 — Supplementary Information [file 41598_2021_85751_MOESM1_ESM.pdf]

SUPPLEMENTARY INFORMATION

A global association between Covid-19 cases and airborne particulate matter at regional levels

Solimini A.[1], Filipponi F. [2], Alunni Fegatelli D. [1], Caputo B. [1], De Marco C.M. [1], Spagnoli A. [1], Vestri AR[1].

1. Department of Public Health and Infectious Diseases, Sapienza University of Rome, Piazzale Aldo Moro 5, 00185 Rome, Italy
2. Institute for Environmental Protection and Research (ISPRA), Via Vitaliano Brancati 48, 00144 Rome, Italy

Supplementary table 1. List of websites from which Covid-19 cases at the date of 1st June, 2020 were retrieved.

Continent	Country	Link to data source
Asia	China	https://raw.githubusercontent.com/CSSEGISandData/COVID-19/master/who_covid_19_situation_reports/who_covid_19_sit_rep_time_series/who_covid_19_sit_rep_time_series.csv
	Japan	https://raw.githubusercontent.com/reustle/covid19japan-data/master/docs/patient_data/2020-04-09.json
	Thailand	https://data.go.th/en/dataset/covid-19-daily
	Indonesia	https://docs.google.com/spreadsheets/d/1ma1T9hWbec1pXlwZ89WakRk-OfVUQZsOCFl4FwZxzVw/edit#gid=2052139453
	South Korea	https://github.com/parksw3/COVID19-Korea/blob/master/COVID19-Korea-2020-04-06.xlsx
Middle East	India	https://raw.githubusercontent.com/mrinal000/covid19_india_data/master/covid19_data/csv/individual/ncov19individual_clean.csv
	Afghanistan	https://docs.google.com/spreadsheets/d/1F-AMEDtqK78EA6LYME2oOsWQsgJi4CT3V_G4Uo-47Rg/export?format=csv
	Pakistan	https://raw.githubusercontent.com/ShahrozTanveer/covid19-pakistan/master/data/covid-19-pakistan-data.csv
	Qatar	https://raw.githubusercontent.com/thlaegler/covid-19-monitor/456bcb1bd4a08963a8acc31529cad64899c52586/data/by_country/Qatar.csv
	Bahrain	https://raw.githubusercontent.com/thlaegler/covid-19-monitor/456bcb1bd4a08963a8acc31529cad64899c52586/data/by_country/Bahrain.csv
	Lebanon	https://raw.githubusercontent.com/thlaegler/covid-19-monitor/456bcb1bd4a08963a8acc31529cad64899c52586/data/by_country/Lebanon.csv
America	United States	https://github.com/nytimes/covid-19-data
	Canada	https://github.com/ishaberry/Covid19Canada/blob/master/cases.csv?raw=true
	Brazil	https://github.com/elhenrico/covid19-Brazil-timeseries/blob/master/confirmed-new.csv
	Chile	https://raw.githubusercontent.com/YachayData/COVID-19/master/COVID19_Chile_Regiones-casos_nuevos.CSV
	Ecuador	https://github.com/pablora19/COVID19_EC/blob/master/covid_ec.csv
	Peru	https://github.com/jmcastagnetto/covid-19-peru-data/blob/master/datos/covid-19-peru-data.csv
	Dominican Republic	https://raw.githubusercontent.com/EmmanuelCruz05/COVID-19_RD/master/COVID-19_RD.csv
	Mexico	https://raw.githubusercontent.com/bialikover/covid-mexico/master/data/confirmados/confirmados-2020-04-05.csv

	Colombia	https://raw.githubusercontent.com/dfuribez/COVID-19-Colombia/master/dataset.csv
	Bolivia	https://raw.githubusercontent.com/mauforonda/covid19-bolivia/master/confirmados.csv
	Paraguay	https://public.tableau.com/profile/mspbs#!/vizhome/COVID19PY-Registros/Descargardatos
	Argentina	https://docs.google.com/spreadsheets/d/16-bnsDdmmgtSxdWbVMboIHo5FRuz76DBxsz_BbsEVWA/export?format=csv&id=16-bnsDdmmgtSxdWbVMboIHo5FRuz76DBxsz_BbsEVWA&gid=0
	Costa Rica	http://geovision.uned.ac.cr/oges/archivos_covid/04_07/04_07_CSV.csv
	Puerto Rico	https://raw.githubusercontent.com/marcmaceira/covid-19-pr-data/master/time_series/municipality/time_series-by_municipality-confirmed.csv
	Cuba	https://raw.githubusercontent.com/covid19cubadata/covid19cubadata.github.io/master/data/covid19-cuba.json
Oceania	Australia	https://raw.githubusercontent.com/pappubahry/AU_COVID19/master/time_series_cases.csv
	New Zealand	https://raw.githubusercontent.com/UoA-eResearch/nz-covid19-data-auto/master/data.csv
Africa	South Africa	https://raw.githubusercontent.com/dsfsi/covid19za/master/data/covid19za_provincial_cumulative_timeline_confirmed.csv
	Algeria	https://raw.githubusercontent.com/dsfsi/covid19africa/master/data/line-list-algeria.csv
	Nigeria	https://raw.githubusercontent.com/dsfsi/covid19africa/master/data/line-list-nigeria.csv
	Ethiopia	https://raw.githubusercontent.com/dsfsi/covid19africa/master/data/line-list-ethiopia.csv
	Ghana	https://raw.githubusercontent.com/dsfsi/covid19africa/master/data/line-list-ghana.csv
	Niger	https://raw.githubusercontent.com/dsfsi/covid19africa/master/data/line-list-niger.csv
	Senegal	https://raw.githubusercontent.com/senegalouvert/COVID-19/master/data/2020.csv
	Gambia	https://raw.githubusercontent.com/dsfsi/covid19africa/master/data/line-list-gambia.csv
Europe	EU-27	https://github.com/ec-jrc/COVID-19/blob/master/data-by-region/jrc-covid-19-all-days-by-regions.csv

Supplementary Figure 1.

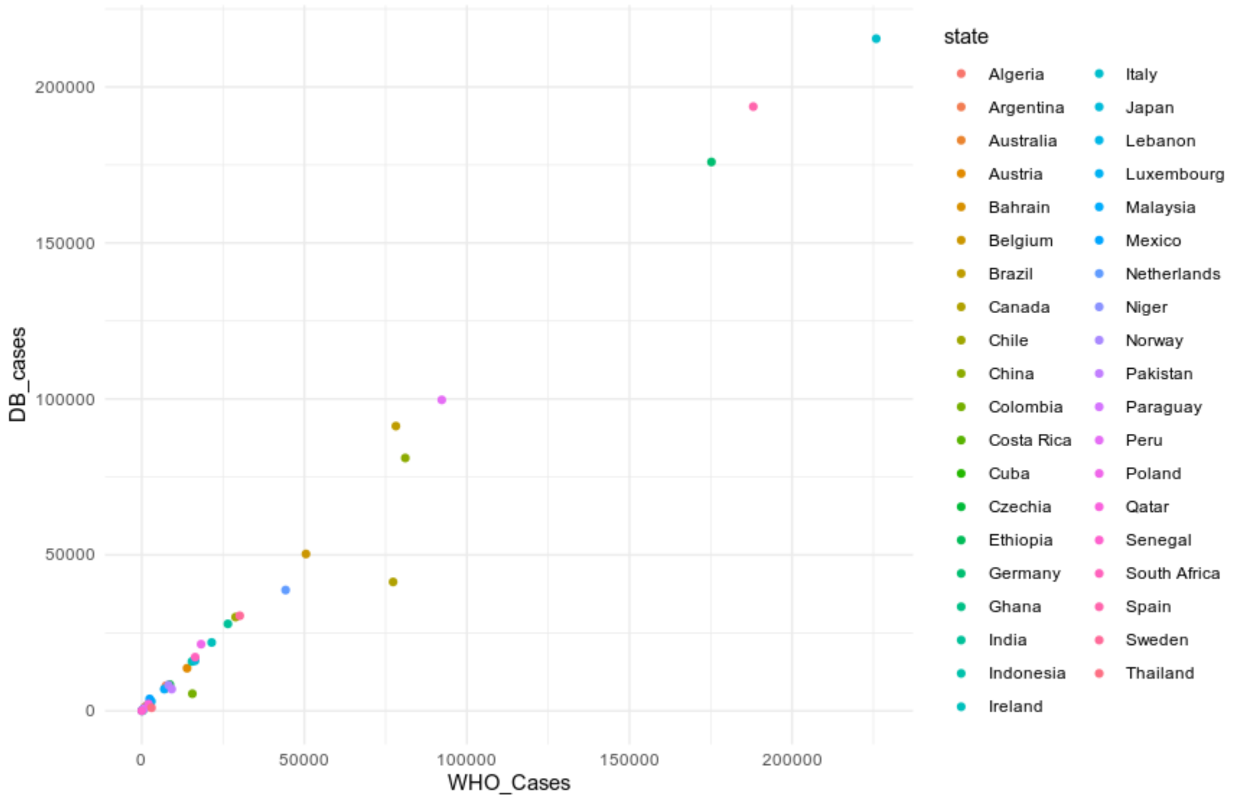

Figure S1. Correlation between Covid cases from various sources by country (as sum over regions) and WHO official data at country level at 1st June, 2020
